# Occult cervical microinvasive squamous cell carcinoma with superficial endometrial spread mimicking high-grade squamous intraepithelial lesion in a postmenopausal woman: a case report

**DOI:** 10.3389/fmed.2026.1881242

**Published:** 2026-07-16

**Authors:** Yuying Chen, Xi Zhao, Gege Wang, Ziwei Liu, Guangchao Sun, Yan Jia

**Affiliations:** 1 Department of Gynecology and Obstetrics, The Second Hospital of Jilin University, Changchun, Jilin, China; 2 Department of General Surgery, The Second Hospital of Jilin University, Changchun, Jilin, China

**Keywords:** cervical high-grade squamous intraepithelial lesion, endometrial involvement, hysterectomy, immunohistochemistry, postmenopausal women, superficial spreading squamous cell carcinoma

## Abstract

**Background:**

Superficial spreading squamous cell carcinoma (SSSCC) of the cervix with endometrial extension is rarely observed in postmenopausal women. Cervical atrophy may reduce the diagnostic accuracy of colposcopy and conization for this condition. We report a case of a subtle microinvasive cervical squamous cell carcinoma with extensive endometrial spread initially misdiagnosed as a high-grade squamous intraepithelial lesion (HSIL) during conization.

**Case presentation:**

We report the case of a 56-year-old postmenopausal woman who was referred after high-risk human papillomavirus positivity and cytology showing atypical squamous cells that could not rule out a high-grade lesion. Colposcopy-directed biopsy and subsequent deep cervical conization showed a high-grade squamous intraepithelial lesion/cervical intraepithelial neoplasia (CIN) grade 3 with glandular involvement, without definite invasive carcinoma. Since residual disease was suspected, she had an extrafascial total hysterectomy (ETH) with bilateral salpingo-oophorectomy. The frozen section revealed squamous neoplasia involving the endometrium. Final pathology showed a cervical high-grade squamous intraepithelial lesion with a microinvasive squamous cell carcinoma focus of less than 0.5 mm, no lymphovascular space invasion (LVSI), superficial spread into the endometrium, and focal superficial invasion. Immunohistochemistry (IHC) showed diffuse block-type p16 positivity, p40 positivity, and negativity for PAX8, estrogen receptor (ER), and progesterone receptor (PR), supporting a cervical origin rather than a primary endometrial squamous cell carcinoma. At 6 months, the patient was disease-free, tested negative for human papillomavirus, and had no intraepithelial lesion or malignancy on cytology.

**Conclusion:**

This case highlights that conization may underestimate occult invasive disease in postmenopausal women with cervical atrophy and glandular involvement. When endometrial squamous lesions are found, superficial spreading cervical squamous neoplasia should be considered, and immunohistochemistry should be performed to distinguish secondary endometrial involvement from primary endometrial disease. For carefully selected stage IA1 lesions without lymphovascular space invasion, extrafascial total hysterectomy can be a definitive treatment option.

## Background

High-grade squamous intraepithelial lesion (HSIL) is the first stage of most cervical squamous cell carcinomas (CSCCs) and is typically treated with excision to eliminate occult invasion and remove the transformation zone (1). In postmenopausal women, however, estrogen deficiency, cervical atrophy, and upward migration of the squamocolumnar junction may compromise colposcopic visualization and reduce the completeness of conization (2). Previous studies have shown that colposcopic assessment is less satisfactory in postmenopausal women and that cold-knife conization is associated with higher rates of positive margins (3, 4). In addition, microinvasive squamous carcinoma may coexist with or be intermingled with extensive HSIL. It is difficult to identify stromal invasion when lesions extend into endocervical glands or the upper cervical canal (5). As a result, the pathology identified in an excision specimen may not fully represent the extent of disease.

A particularly unusual pattern is superficial spreading squamous cell carcinoma (SSSCC) of cervical origin. In this variant, the neoplastic squamous epithelium extends proximally along the endometrium rather than presenting as deep stromal invasion or distant metastasis. Since the first description of endometrial involvement by cervical squamous neoplasia in 1971, only a few such cases have been reported (2, 6, 7). The lesion may mimic a primary endometrial process, especially in postmenopausal women with an atrophic uterine cavity.

We present a postmenopausal patient whose conization showed only HSIL with glandular involvement, whereas hysterectomy disclosed occult stage IA1 CSCC with diffuse superficial endometrial spread. Compared with previous cases, the educational value of this case lies in the striking discrepancy between the conization diagnosis and the hysterectomy findings, as well as the diagnostic challenge posed by the atrophic postmenopausal cervix. It also highlights the central role of immunohistochemistry (IHC) in confirming the cervical origin of the lesion and excluding primary endometrial squamous carcinoma.

## Case presentation

A 56-year-old woman, who was 7 years postmenopausal, presented in October 2025 after screening revealed positivity for high-risk human papillomavirus (HPV) 16 and 18, and other high-risk types. ThinPrep cytology showed atypical squamous cells that could not exclude HSIL. Colposcopy-directed biopsy showed HSIL/cervical intraepithelial neoplasia (CIN) grade 3 with endocervical gland involvement.

Since the patient strongly wished to preserve her uterus, a deep and wide cervical conization was performed. Histopathologic examination of the cone specimen showed HSIL/CIN 3 involving the cervical and endocervical tissue with glandular extension, but no definite invasion was identified. Given the patient’s postmenopausal status and concern for residual or occult disease, definitive surgery was discussed. One month later, she consented to an open extrafascial total hysterectomy (ETH) with bilateral salpingo-oophorectomy.

**Figure 1:**
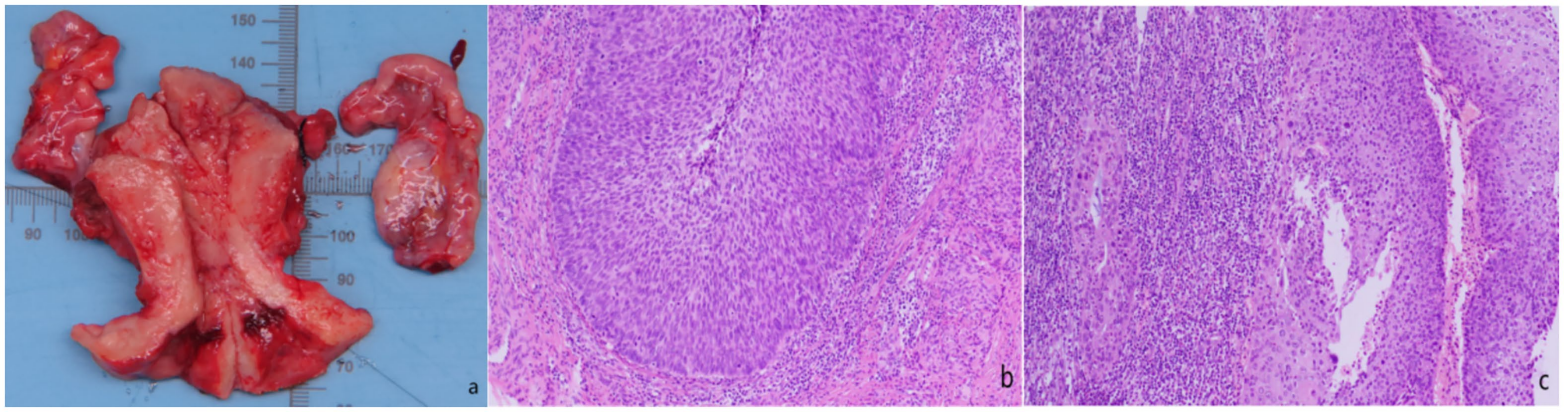
Cervical lesion and uterine specimen. **(a)** Gross view of the hysterectomy specimen with opened endometrial cavity. **(b)** HSIL/CIN3 involving endocervical glands (HE, ×100). **(c)** Microinvasive squamous cell carcinoma arising in HSIL (HE, ×100).

**Figure 2:**
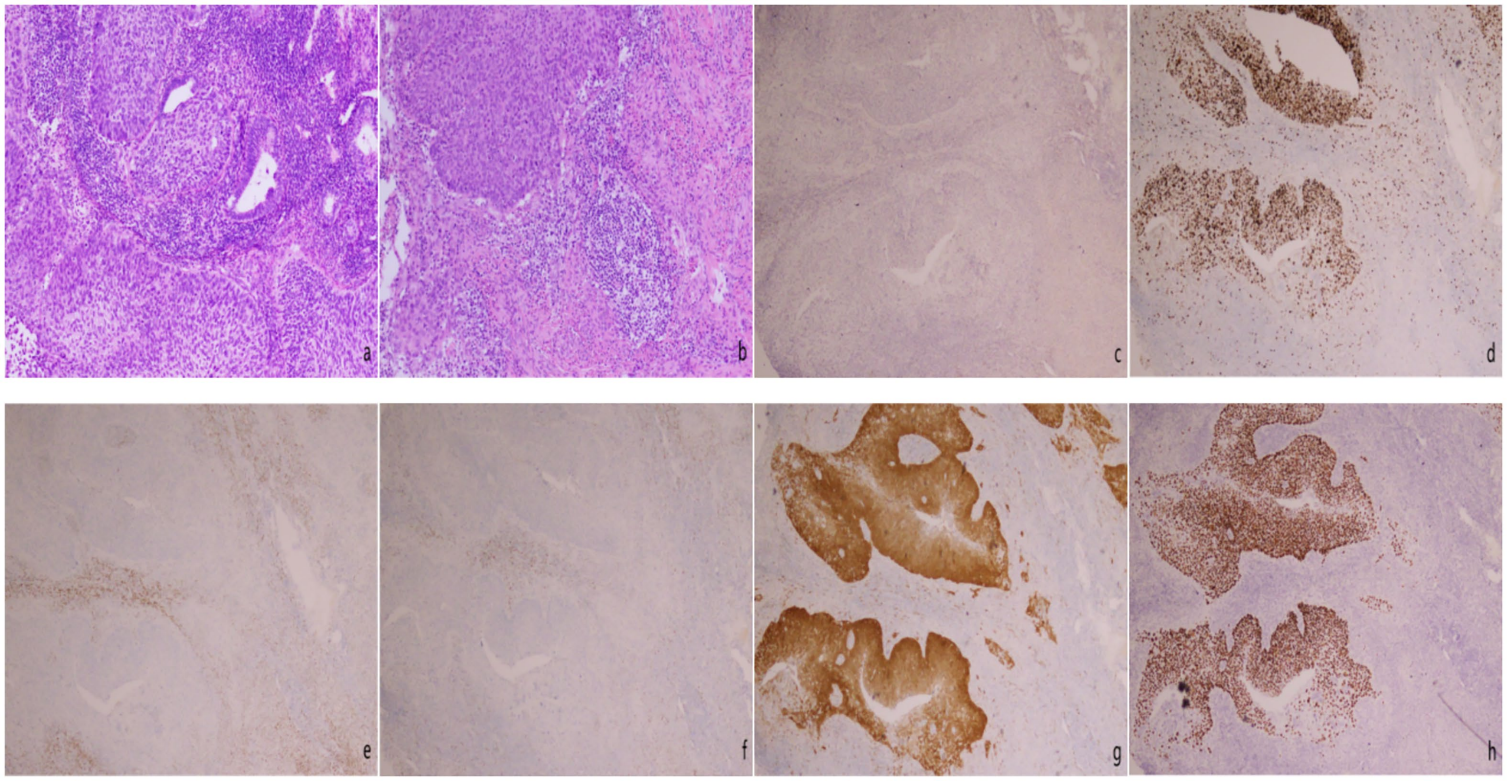
Superficial endometrial spread of cervical squamous neoplasia. **(a)** HSIL replacing the endometrial surface (HE, ×100). **(b)** Superficial myometrial invasion by well-differentiated squamous cell carcinoma (HE, ×100). **(c)** PAX8-negative tumor cells with positive internal control in benign endometrial glands (×100). **(d)** Ki-67 proliferation index ~80% (×100). **(e)** ER negativity in tumor cells (×100). **(f)** PR negativity in tumor cells (×100). **(g)** Diffuse block-type p16 staining (×100). **(h)** p40 positivity confirming squamous differentiation (×100).

At surgery, the endometrial surface appeared rough and firm (see Figure 1a). The hysterectomy specimen was formalin-fixed, paraffin-embedded, and examined using hematoxylin and eosin (HE) staining. The frozen section identified squamous neoplasia involving the endometrium with focal carcinoma. Permanent sections confirmed the following: (1) cervical HSIL with glandular involvement and a focus of microinvasive squamous cell carcinoma measuring less than 0.5 mm in depth (see Figures 1b,c), without lymphovascular space invasion (LVSI); (2) extensive involvement of the endometrial surface by HSIL and moderately differentiated squamous cell carcinoma (see Figures 2a,b); and (3) focal superficial myometrial invasion beneath the endometrial lesion.

IHC performed on representative tissue sections showed diffuse strong p16 expression, p40 positivity, PAX8 negativity, estrogen receptor negativity, progesterone receptor negativity, Ki-67 labeling index of approximately 80% (see Figures 2c–h), p53 positivity, CDX2 negativity, and membranous *β*-catenin staining. The shared squamous immunophenotype in the cervix and endometrium supported a single cervical primary with secondary superficial spread to the uterine cavity, rather than a primary endometrial squamous cell carcinoma or endometrial adenocarcinoma with squamous differentiation.

The postoperative course was uneventful. At the 6-month follow-up, high-risk HPV testing was negative, and repeat cytology showed no intraepithelial lesion or malignancy.

## Discussion

This case highlights that conization may underestimate the true extent of cervical squamous neoplasia in postmenopausal women. Cervical atrophy, stenosis of the external os, and recession of the transformation zone into the endocervical canal can create a diagnostic blind area, even when a deep excision is performed (2, 4). Prior studies have shown lower colposcopic adequacy and higher positive-margin rates after conization in postmenopausal women (8). In addition, invasive squamous carcinoma may arise in the background of extensive HSIL, and small foci of stromal invasion may be obscured by glandular involvement or sampling limitations (9). In this patient, the cone specimen showed only HSIL, whereas hysterectomy revealed occult microinvasive carcinoma and extensive proximal spread. Glandular involvement and an unsatisfactory or high endocervical transformation zone should therefore raise concern that conization may sample only the most accessible portion of the disease.

Furthermore, superficial spreading squamous neoplasia of cervical origin should be considered when squamous lesions are identified in the endometrium. Previous studies have suggested that tumor cells may extend along the mucosal surface continuously, replacing the atrophic endometrium and occasionally infiltrating the superficial myometrium (6, 7, 10). This pattern may be more likely to occur in the postmenopausal uterine cavity due to the thin endometrium and reduced resistance at the internal os. In our case, the discordance between the small cervical invasive focus and the widespread endometrial involvement could easily have suggested a primary endometrial lesion had the cervical history not been carefully integrated into the pathologic interpretation.

Third, intraoperative frozen section examination was very useful in this case. It identified squamous tumors and focal canceration in the endometrium and enhanced the adequate sampling of the uterus after the operation. However, they cannot determine the source of uterine squamous lesions and rely on permanent sections and IHC. IHC played a crucial role in the differential diagnosis. The diagnosis of primary endometrial squamous cell carcinoma is exceptionally rare and should be made only after ruling out a cervical primary and endometrial adenocarcinoma with squamous differentiation (11). In this patient, diffuse p16 and p40 positivity, together with negativity for PAX8, estrogen receptor, and progesterone receptor, strongly indicated cervical squamous neoplasia with secondary endometrial spread rather than a primary *Müllerian-dervived tumor* (12, 13). This integrated morphologic and immunophenotypic approach is essential for evaluating uterine squamous lesions.

From a management perspective, the cervical lesion met criteria for stage IA1 disease because stromal invasion was less than 0.5 mm and no LVSI was identified (14, 15). Although the hysterectomy specimen showed uterine corpus involvement, this does not in itself upstage cervical cancer in the current International Federation of Gynecology and Obstetrics staging system (15). For stage IA1 cervical squamous carcinoma without LVSI, ETH is an acceptable treatment option when fertility preservation is not required (14). In this case, the favorable primary cervical pathology and complete resection supported against more radical surgery or adjuvant radiotherapy. The patient’s negative 6-month surveillance results provide preliminary evidence of disease control. Due to the short follow-up, these results must be interpreted cautiously, and longer follow-up is required to evaluate sustained remission and recurrence risk.

Compared with recently published reports, this case focuses on the inconsistency between pathological diagnosis results after conization and hysterectomy, as well as the differential diagnosis of SSSCC. Jiang et al. have summarized the clinicopathological characteristics of superficial spreading cervical squamous lesions (10). In addition, Song et al. have reported an elderly patient with an intrauterine abscess in which the SSSCC lesion spread to the fallopian tube mucosa (7). Chitturi et al. have described a case in which magnetic resonance imaging (MRI) showed that the lesion had spread to the uterus and demonstrated obvious interstitial infiltration (2). In our case, the deepened and widened conotomy specimens showed HSIL with glandular involvement. However, hysterectomy revealed a concealed stage IA1 microinvasive squamous cell carcinoma accompanied by extensive superficial endometrial spread. This indicates that the scope of preoperative sampling is still limited and may not be able to capture the full extent of the disease. Additionally, it is necessary to use IHC to distinguish between primary and secondary lesions. There are several limitations in this case report. First, the follow-up period of 6 months is not sufficient to assess the risk of long-term recurrence, and continuous monitoring is required. Second, ascites cytology was not performed because the preoperative diagnosis considered residual HSIL or latent early cervical lesions, rather than primary endometrial cancer or advanced cervical cancer. Therefore, ascites was not collected as required by the tumor stage. However, due to a lack of this information, we are unable to provide an objective assessment of the abdominal cavity dissemination status. Third, since the diffusion pattern of SSSCC is extremely rare, preoperative imaging examinations have not been designed for it. In retrospect, a specialized pelvic MRI might provide more information about insidious spread into the uterine cavity. However, in a conization or postmenopausal atrophied uterus, small cervical infiltrative lesions and superficial spreading mucosal lesions may still not be radiologically evident (16).

## Conclusion

Occult cervical microinvasive squamous cell carcinoma may be interpreted as HSIL during conization and spread superficially throughout the endometrium in postmenopausal women. Awareness of this rare spread pattern, attention to postmenopausal sampling limitations, and application of a targeted IHC panel can improve diagnostic accuracy and help avoid undertreatment and overtreatment.

## Data Availability

The original contributions presented in the study are included in the article/supplementary material, further inquiries can be directed to the corresponding author.
